# Extraction and complexation studies with 2,6-bis(5-(*tert*-butyl)-1*H*-pyrazol-3-yl)pyridine in the presence of 2-bromohexanoic acid†

**DOI:** 10.1039/d4ra05630b

**Published:** 2024-09-05

**Authors:** Jonas Stracke, Patrik Weßling, Thomas Sittel, Paul Meiners, Andreas Geist, Petra J. Panak.

**Affiliations:** a Karlsruhe Institute of Technology (KIT), Institute for Nuclear Waste Disposal (INE) P.O. Box 3640 76021 Karlsruhe Germany Jonas.stracke@kit.edu; b Heidelberg University, Institut für Physikalische Chemie Im Neuenheimer Feld 253 69120 Heidelberg Germany

## Abstract

To improve the understanding of the extraction chemistry of An(iii) and Ln(iii) with N-donor ligands 2,6-bis(5-(*tert*-butyl)-1*H*-pyrazol-3-yl)pyridine (C4-BPP) in the presence of 2-bromohexanoic acid was investigated. Extraction studies showed an excellent separation factor of SF_Am(III)/Eu(III)_ ≈ 200 and SF_Am(III)/Nd(III)_ ≈ 60 in comparison with the structurally similar ligand 2,6-bis(5-neopentyl-1*H*-pyrazol-3-yl)pyridine C5-BPP (SF_Am(III)/Eu(III)_ ≈ 100), even though C5-BPP showed significantly higher stability constants. Time-resolved laser fluorescence spectroscopy (TRLFS) studies revealed the formation of the ternary 1 : 1 and 1 : 2 complexes [Eu(C4-BPP)_*n*_(2-bromohexanoate)_*m*_]^(3−*m*)+^ (*n* = 1–2) (
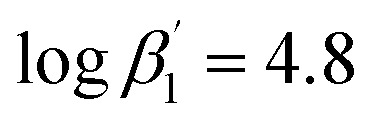
 and 
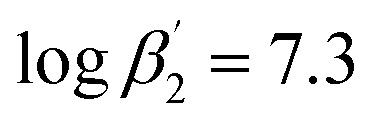
). [Eu(C4-BPP)_2_(2-bromohexanoate)_*m*_]^(3−*m*)+^ was the relevant complex species in solvent extraction. In contrast, Cm(iii) form stable 1 : 3 complexes. The ability of 2-bromohexanoic acid to replace C4-BPP from the inner coordination sphere of Eu(iii) but not from Cm(iii) is due to a more favorable complexation of Cm(iii) over Eu(iii) with C4-BPP. This resulted in a notably more efficient separation of An(iii) and Ln(iii) in comparison with C5-BPP.

## Abstract 2

Extraction studies of An(iii) and Ln(iii) with C4-BPP showed an excellent separation factor of SF_Am(__iii__)/Eu(iii)_ ≈ 200 due to [Eu(C4-BPP)_2_(2-bromohexanoate)*_m_*]^(3−*m*)+^ (*m* = 1–3). In contrast, Cm(iii) forms significantly more stable homoleptic complexes.

## Graphical abstract

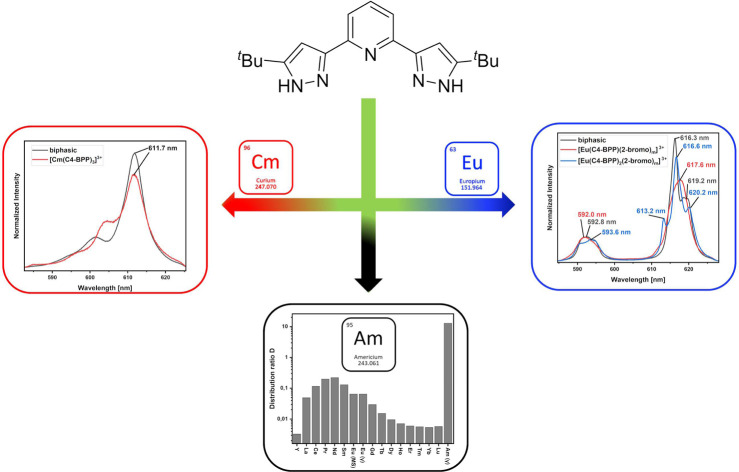

## Introduction

Multi-recycling of irradiated nuclear fuel would significantly reduce the environmental footprint of nuclear energy since the required amount of uranium to be mined and milled would be reduced drastically. Instead, fresh MOX (Mixed Oxide Fuel) fuel would be fabricated from depleted or reprocessed uranium and reprocessed plutonium, to be used in reactors with a fast neutron spectrum. Additionally, recycling americium would furthermore reduce the long-term heat load of the high level waste to be finally disposed of in a deep geologic repository, significantly increasing its capacity.^1–6^ While recovering uranium and plutonium is performed on an industrial level applying the PUREX (Plutonium Uranium Reduction EXtraction) process, recovering Am(iii) is so far studied only in the laboratory. Am(iii) must be separated from the fission lanthanides, which is challenging due to their chemical similarity.^7–9^ However, the required selectivity can be achieved by solvent extraction using ligands with soft donor atoms such as nitrogen.^10,11^*E.g.*, bis(triazinyl)pyridines (BTPs) or bis(triazinyl)bipyridines (BTBPs) have proven to be highly efficient extracting agents to separate An(iii) from Ln(iii).^12,13^

**Fig. 1:**
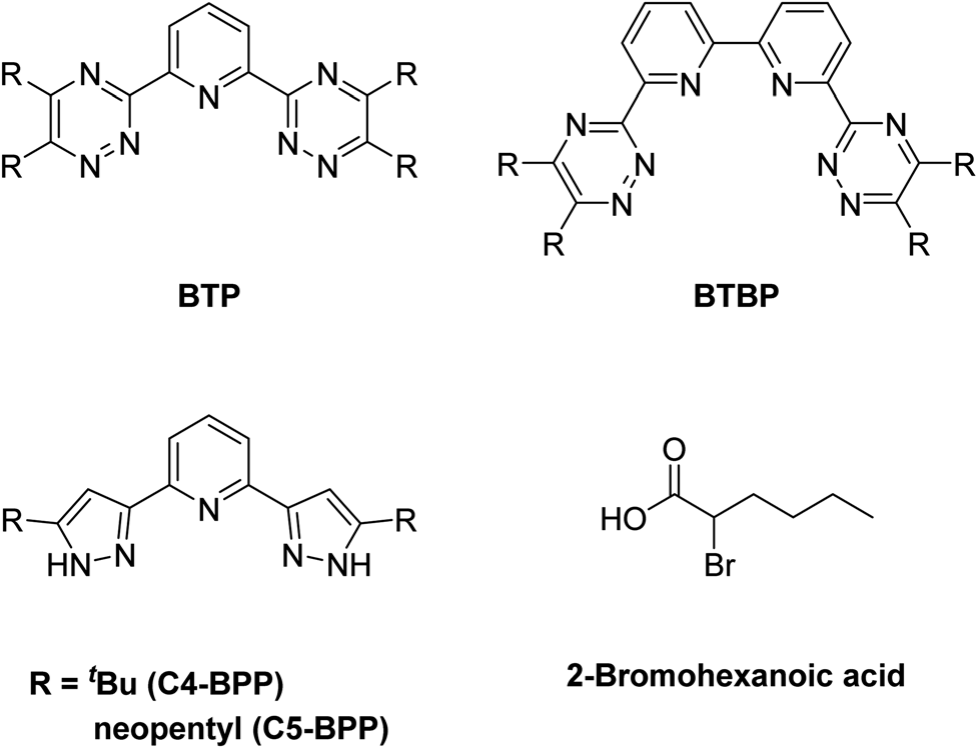
General structural motifs of BTP (top left), BTBP (top right), BPP (bottom left) and structure of 2-bromohexanoic acid (bottom right).

In addition to detailed studies on BTPs and BTBPs, numerous ligands are being investigated to elucidate their selectivity by systematical variation of their aromatic backbones.^12,14–20^ For example, the triazinyl groups of the BTP backbone have been replaced with pyrazolyl rings in the ligand 2,6-bis(5-(2,2-dimethylpropyl)-1*H*-pyrazol-3-yl)pyridine (C5-BPP) (Fig. 1).^21^ C5-BPP shows a good performance as a selective extracting agent^21^ and the Cm(iii) 1 : 3 complex 
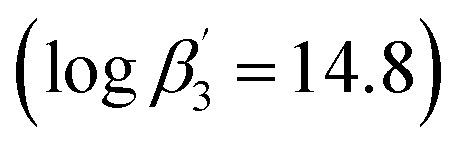
^21^ has a similar stability constant compared to the Cm(iii) 1 : 3 complex with BTP 
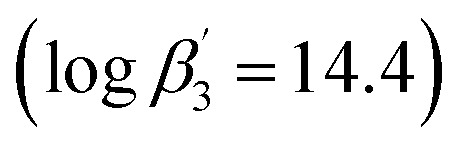
.^22^. Nonetheless, C5-BPP requires, in contrast to BTPs and BTBPs, a lipophilic anion source, 2-bromohexanoic acid (Fig. 1), to selectively extract Am(iii) from nitric acid solutions.^23^ This indicates, that extracting abilities might not only result from sheer complexation strength and makes it worthwhile to study this topic even further. Since variation of the sidechain may result in large deviations of the properties of these ligands, for example *^n^*Pr-BTP and *^i^*Pr-BTP,^24–26^ C4-BPP (Fig. 1) was investigated thereupon. Complexation studies have shown the formation of [Cm(C4-BPP)_*n*_]^3+^ (*n* = 1–3) and [Eu(C4-BPP)_*n*_]^3+^ (*n* = 1–2) complexes.^27^ The stability constant of the Cm(iii) 1 : 3 complex with C4-BPP 
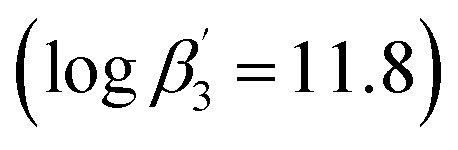
^27^ is lower than the one with C5-BPP 
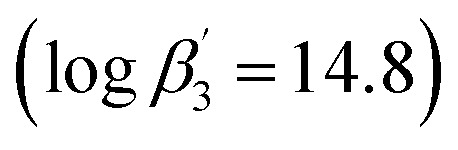
,^21^ which can be attributed to the steric demand of the ^*t*^Bu moieties compared to the neopentyl groups. Now the focus is on the effect of these differences on solvent extraction properties and to determine the composition of the extracted species. Since C5-BPP requires a lipophilic anion source,^21^ 2-bromohexanoic acid (Fig. 1), it is expected that C4-BPP also needs an additional anion source.

## Experimental section

### Liquid–liquid extraction

The performance of C4-BPP as an extracting agent is determined by the distribution ratios (*D* = *c*_org_/*c*_aq_) of ^241^Am(iii), ^152^Eu(iii), Y(iii) and the Ln(iii) series (except Pm(iii)). The organic phase consists of 1.0 × 10^−2^ mol per L C4-BPP and 5.0 × 10^−1^ mol per L 2-bromohexanoic acid dissolved in TPH (tetrapropylhydrogène; main component: 1,1,2,2,3,3,4,4-octamethylbutyl) with 10 vol% of 1-octanol. As aqueous phase HNO_3_ (0.05–1.00 M) is used, which is spiked with each 1 kBq/mL ^241^Am(iii), ^152^Eu(iii) and 20 mg per L Y(iii), La(iii)–Lu(iii) (except Pm(iii)) as nitrate salts. Equal volumes (500 μL) of organic and aqueous phases (A/O (Aqueous/Organic) = 1) are contacted for 30 min at 293 K using an orbital shaker (2500 rpm). It has been tested previously that 30 min are sufficient to reach equilibrium. Samples are centrifuged to separate the phases. 300 μL of each phase are analyzed on a gamma counter (Packard Cobra Auto-Gamma 5003). For ICP-MS (Inductively coupled plasma mass spectrometry) organic phases are stripped with 0.5 mol per L ammonium glycolate solution (pH = 4, A/O = 10) and further diluted with 2% ultrapure HNO_3_. Aqueous phases are diluted with 2% ultrapure HNO_3_.

### TRLFS (time-resolved laser-induced fluorescence spectroscopy) sample preparation

Stock solutions of C4-BPP are prepared by dissolving 85 mg in 1200 μL of methanol containing 1.5 vol% of water (2.2 × 10^−1^ mol L^−1^). Solutions with lower C4-BPP concentrations are obtained through dilution. Cm(iii)-TRLFS samples are prepared by adding 4.7 μL of a Cm(iii) stock solution (2.12 × 10^−5^ mol per L Cm(ClO_4_)_3_ in 1.0 × 10^−1^ mol per L HClO_4_; ^248^Cm: 89.7%, ^246^Cm: 9.4%, ^243^Cm: 0.4%, ^244^Cm: 0.3%, ^245^Cm: 0.1%, ^247^Cm: 0.1%) to 985 μL of methanol and 10.3 μL of water, resulting in an initial Cm(iii) concentration of 1.0 × 10^−7^ mol L^−1^. Eu(iii)-TRLFS samples are prepared by adding 9.4 μL of a Eu(iii) stock solution (1.07 × 10^−3^ mol per L Eu(ClO_4_)_3_ in 1.0 × 10^−2^ mol per L HClO_4_ to 985 μL of methanol (or 914 μL of methanol and 71 μL; 97.6 mg 2-bromohexanoic acid) and 5.6 μL of water, resulting in an initial Eu(iii) concentration of 1.0 × 10^−5^ mol L^−1^. By adding appropriate volumes of the various ligand solutions different ligand concentrations are adjusted. The resulting solutions are allowed to equilibrate for 10 min before measurement to reach chemical equilibrium.

### TRLS measurements

TRLFS measurements are performed at 293 K using a Nd:YAG (Surelite II laser, Continuum) pumped dye laser system (NarrowScan D-R; Radiant Dyes Laser Accessories GmbH). Cm(iii) is exited at a wavelength of 396.6 nm, Eu(iii) at a wavelength of 394.0 nm. A spectrograph (Shamrock 303i, ANDOR) with 300, 1199 and 2400 lines per millimeter gratings is used for spectral decomposition. The detection of fluorescence emission is performed using an ICCD camera (iStar Gen III; ANDOR). To discriminate short-lived organic fluorescence and light scattering a delay time of 1 μs is set.

## Results and discussion

### Liquid–liquid extraction

To study the extraction properties of C4-BPP, distribution ratios of An(iii) and Ln(iii) are determined as a function of nitric acid concentration. A lipophilic anion source, 2-bromohexanoic acid, is added as C4-BPP is not able to extract nitrates from nitric acid solutions. The extraction of metal ions follows a cation-exchange mechanism instead (1).^21,28,29^

1 M_aq._^3+^ + 3HA_org_ + *n*C4-BPP ⇌ [M(C4-BPP)_*n*_][A_3_]_org_ + 3H_aq._^+^ (*n* = 1–3)

**Fig. 2:**
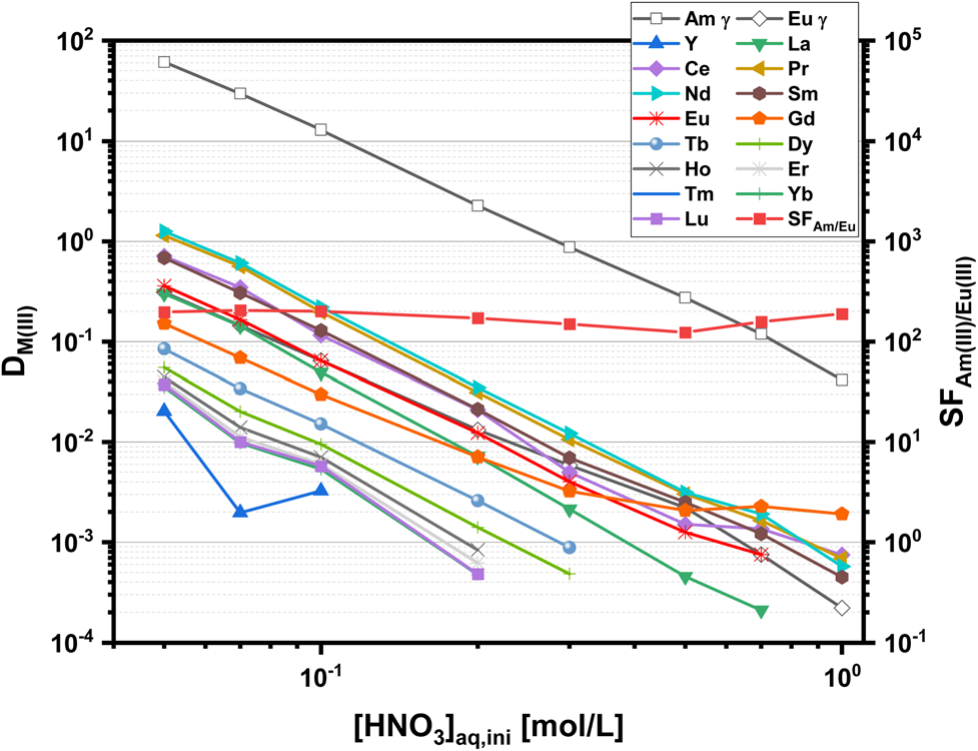
Distribution ratios for the extraction of Am(iii) and Eu(iii) (γ, open symbols) and Ln(iii) (ICP-MS, solid symbols) with C4-BPP. Organic phase 1.0 × 10^−2^ mol L^−1^ C4-BPP and 5.0 × 10^−1^ mol L^−1^ 2-bromohexanoic acid in TPH/1-octanol (10 vol%). Aqueous phase 5.0 × 10^−2^–1.00 mol L^−1^ nitric acid. A/O = 1. *T* = 293 K.

The dependence of distribution ratios on nitric acid concentrations is shown in Fig. 2.

**Fig. 3:**
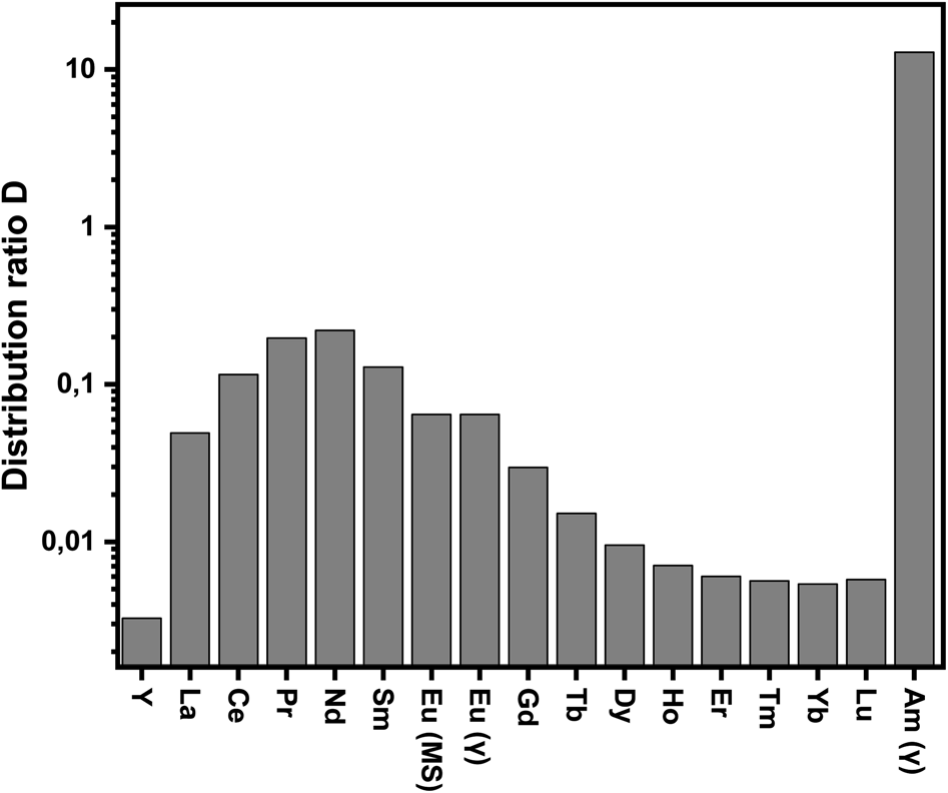
Distribution ratios for the extraction of Am(iii) and Ln(III) with C4-BPP. Organic phase 1.0 × 10^−2^ mol L^−1^ C4-BPP and 5.0 × 10^−1^ mol L^−1^ 2-bromohexanoic acid in TPH/1-octanol (10 vol%). Aqueous phase 1.0 × 10^−1^ mol L^−1^ nitric acid. A/O = 1. *T* = 293 K.

Distribution ratios for all metal ions decrease with increasing nitric acid concentration. This is in agreement with a cation exchange mechanism according to (1). With an organic phase comprising 10 mmol L^−1^ C4-BPP and 5.0 × 10^−1^ mol L^−1^ 2-bromohexanoic acid in a TPH/1-octanol diluent (9 : 1 vol), Am(iii) is extracted from HNO_3_ solutions with concentrations <0.3 mol L^−1^ while Ln(iii) remain in the aqueous phase. Am(iii) back extraction is achieved at HNO_3_ concentrations >0.3 mol L^−1^. All Ln(iii) are hardly extracted, with Nd(iii) showing the highest distribution ratio among the Ln(iii) series. Nevertheless, a separation factor of SF_Am(III)/Nd(III)_ ≈ 60 allows efficient separation of Am(iii) from Nd(iii). For the remaining Ln(iii) separation factors up to SF_Am(III)/Eu(III)_ ≈ 200 are achieved. These results fit perfectly with literature data, proving a highly favored complexation of An(iii) over Ln(iii).^27^ The slopes for log *D*_Am(III)_ and log *D*_Eu(III)_*vs.* [HNO_3_] are −2.4 and −2.3, respectively, which differ slightly from the expected value of three. This effect can be attributed to the presence of 1-octanol in the diluent as observed previously for C5-BPP.^30^ Distribution ratios of all Ln(iii), with exception of Pm(iii), at a constant nitric acid concentration (0.1 mol L^−1^) are shown in Fig. 3. Compared to C5-BPP distribution ratios follow the same trend for Am(iii) and the Ln(iii).^29^ Despite this similarity C4-BPP shows a somewhat higher separation efficiency than C5-BPP (SF_Am(III)/Eu(III)_ ≈ 100).

**Fig. 4:**
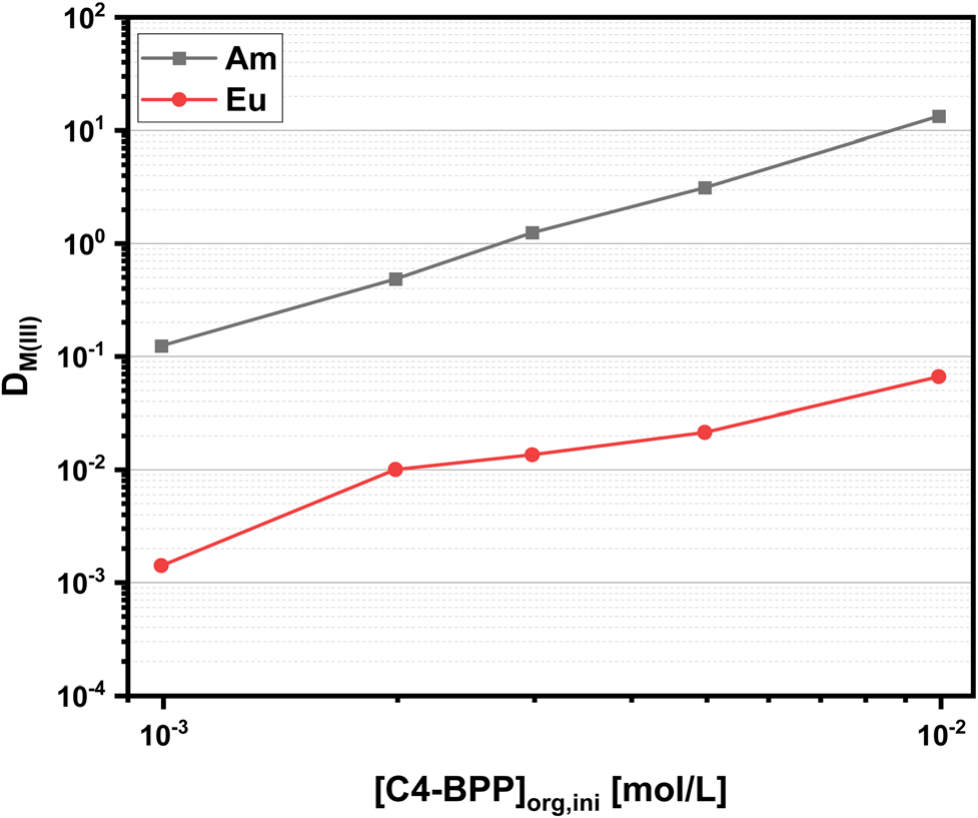
Distribution ratios for the extraction of Am(iii) and Eu(iii) with C4-BPP. Organic phase 1.0 × 10^−3^–1.0 × 10^−2^ mol L^−1^ C4-BPP and 5.0 × 10^−1^ mol L^−1^ 2-bromohexanoic acid in TPH/1-octanol (10 vol%). Aqueous phase 1.0 × 10^−1^ mol L^−1^ nitric acid. A/O = 1. *T* = 293 K.

To further investigate the extraction performance of C4-BPP, Am(iii) and Eu(iii) distribution ratios as a function of the C4-BPP concentration are determined. The distribution ratios are shown in Fig. 4.

The distribution ratios increase with increasing C4-BPP concentration, with the separation factor increasing from around SF_Am(III)/Eu(III)_ ≈ 90 (1.0 × 10^−3^ mol L^−1^) to SF_Am(III)/Eu(III)_ ≈ 200 (1.0 × 10^−2^ mol L^−1^). The slopes for log *D*_Am(III)_ and log *D*_Eu(III)_*vs.* [C4-BPP] are 2.0 and 1.6 respectively, which indicates the formation of [M(C4-BPP)_2_]^3+^. This is in good agreement with the results for C5-BPP, which also show slopes of less than two. These results indicated self-association and/or association of the ligand with 2-bromohexanoic acid.^30^ Since former complexation studies by TRLFS show the formation of [Cm(C4-BPP)_3_]^3+^, it can be assumed that self-association undermines the efforts to determine the number of coordinated C4-BPP molecules in the extracted species by slope analysis.^27^

**Fig. 5:**
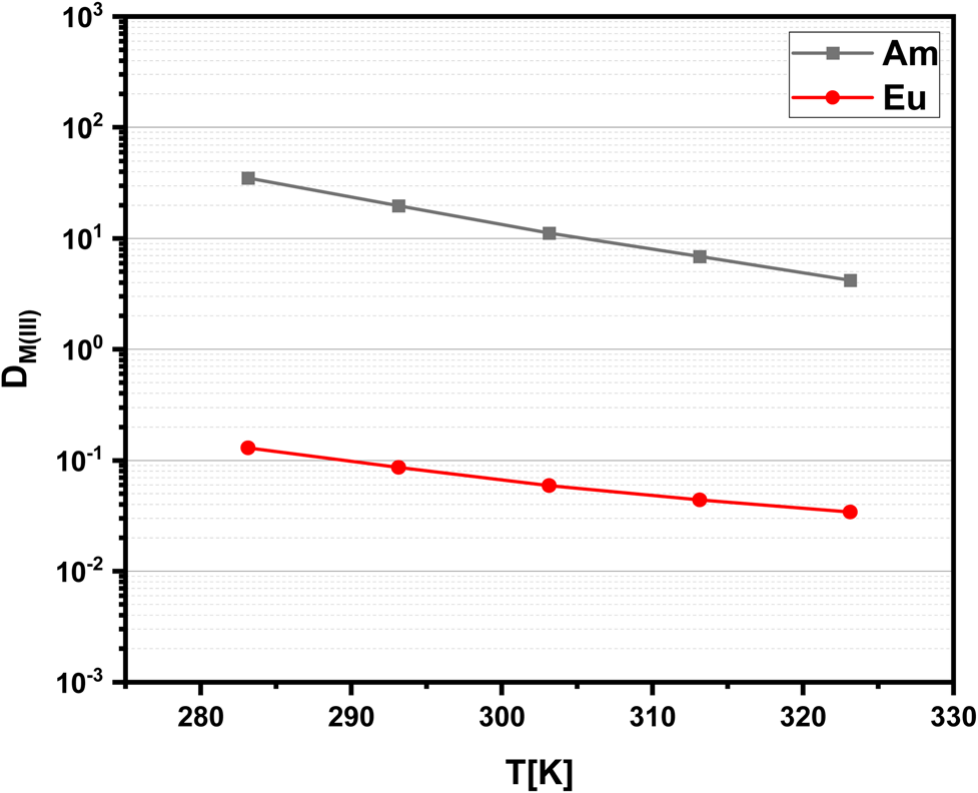
Distribution ratios for the extraction of Am(iii) and Eu(iii) with C4-BPP as a function of the temperature organic phase 1.0 × 10^−2^ mol L^−1^ C4-BPP and 5.0 × 10^−1^ mol L^−1^ 2-bromohexanoic acid in TPH/1-octanol (10 vol%). Aqueous phase 1.0 × 10^−1^ mol L^−1^ nitric acid. A/O = 1. *T* = 283–323 K.

Since solvent extraction processes are not necessarily performed at a temperature of 293 K, distribution ratios of Am(iii) and Eu(iii) are determined at a constant nitric acid concentration as a function of temperature. The results are shown in Fig. 5. With increasing temperature, a decrease of the distribution ratios is observed, which indicates an exothermic reaction. Further has to be noted, that the slope is steeper for Am(iii) compared to Eu(iii), resulting in decreasing separation factors with increasing temperatures SF_Am(III)/Eu(III)_ ≈ 110 (323 K).

### Speciation of Cm(iii) and Eu(iii) in the solvent extraction samples

**Fig. 6:**
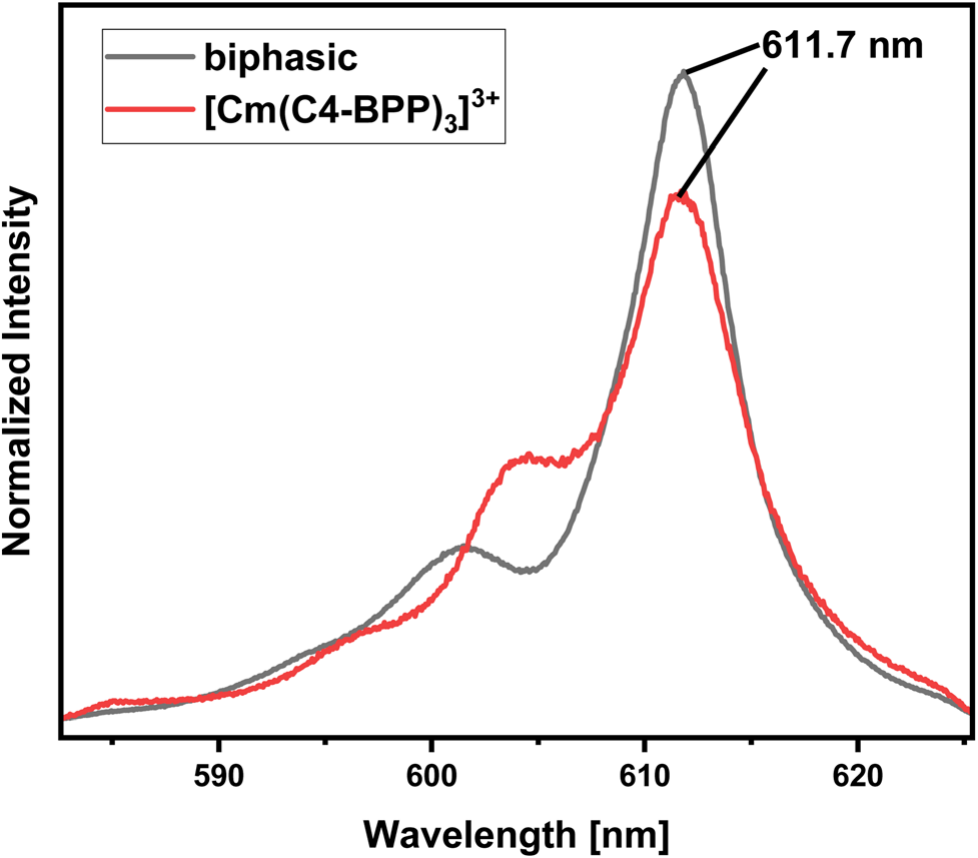
Normalized fluorescence spectra of Cm(iii) in the organic phase after extraction (black) and the [Cm(C4 BPP)_3_]^3+^ complex (red) in methanol containing 1.5 vol% water. Organic phase 1.0 × 10^−2^ mol L^−1^ C4-BPP and 5.0 × 10^−1^ mol L^−1^ 2-bromohexanoic acid in TPH/1-octanol (10 vol%). Aqueous phase 3.0 × 10^−1^ mol L^−1^ nitric acid. A/O = 1. *T* = 293 K.

To investigate the speciation under extraction conditions (0.3 mol L^−1^ nitric acid and 0.5 mol L^−1^ 2-bromohexanoic acid), both phases of a solvent extraction experiment are examined by TRLFS. The organic phase containing Cm(iii) (Fig. 6, biphasic) shows an emission spectrum with a maximum at 611.7 nm, displaying exactly the same spectrum as in the titration experiment for the [Cm(C4-BPP)_3_]^3+^ complex.^27^ This confirms the coordination of three C4-BPP molecules and thus the absence of 2-bromohexnoic acid in the inner coordination sphere. The deviation in shape of the spectra may result from the different solvents (TPH *vs.* methanol). The complex shows a fluorescence lifetime of 413 ± 20 μs (ESI, Fig. S1†). The emission spectrum of the aqueous phase shows two maxima at 594 nm and 596.7 nm (ESI, Fig. S2†), which are assigned to [Cm_aq_]^3+^ and [Cm(NO_3_)]^2+^, respectively.^31,32^ Furthermore, the fluorescence lifetime of 73 ± 4 μs (ESI, Fig. S3†) is located between 68 μs ([Cm_aq_]^3+^) and 82 μs ([Cm(NO_3_)]^2+^), which shows the presence of both species.^33,34^

**Fig. 7:**
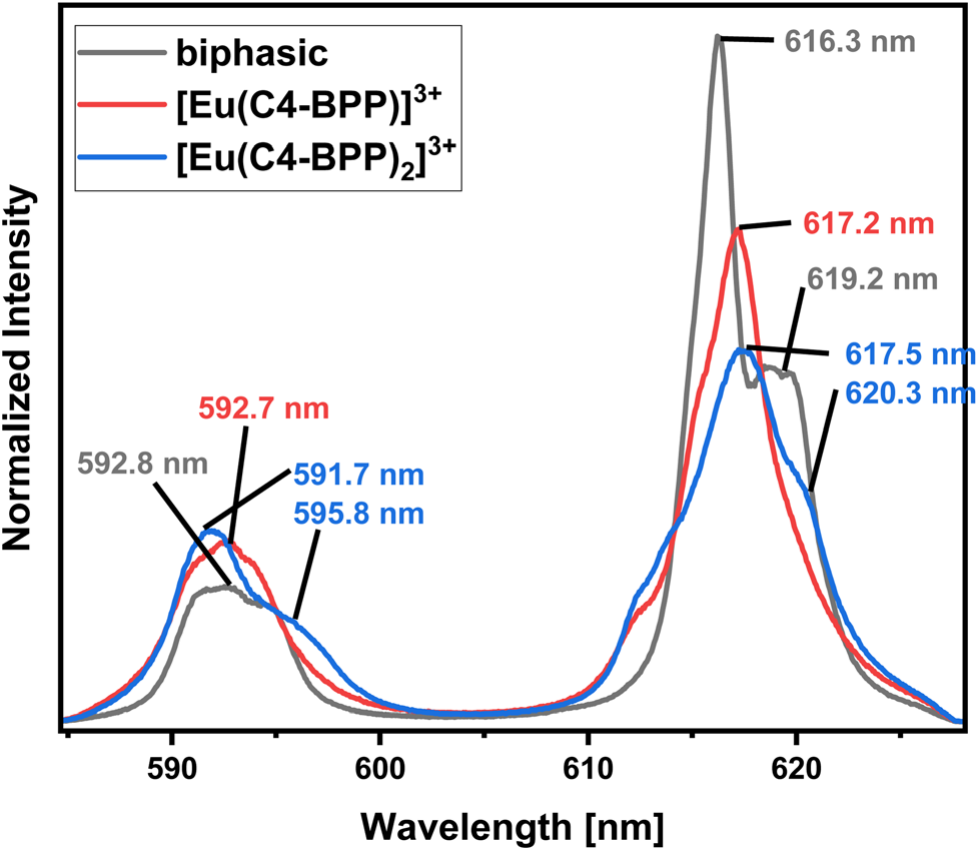
Normalized fluorescence spectra of the ^7^F_1_ and ^7^F_2_ emission bands of Eu(iii) in the organic phase after extraction (black), [Eu(C4 BPP)]^3+^ complex (red) and [Eu(C4 BPP)_2_]^3+^ complex (blue) in methanol containing 1.5 vol% water. Organic phase 1.0 × 10^−2^ mol L^−1^ C4-BPP and 5.0 × 10^−1^ mol L^−1^ 2-bromohexanoic acid in TPH/1-octanol (10 vol%). Aqueous phase 3.0 × 10^−1^ mol L^−1^ nitric acid. A/O = 1. *T* = 293 K.

In contrast to Cm(III), the emission bands of Eu(III) obtained from the organic phase (Fig. 7, biphasic) differ significantly from those of the titration experiment and cannot be assigned to any of the formerly observed complexes.^27^ However, the fluorescence lifetime of 1314 ± 66 (ESI, Fig. S4†) shows the absence of water molecules in the first coordination sphere.^35^ Therefore, the coordination of either 2-bromohexanoic acid or nitrate was assumed. Considering the more lipophilic properties of 2-bromohexanoic acid compared to nitrate, its participation seems more likely. The emission spectrum of the aqueous phase shows maxima at 592.0 nm (^5^D_0_ → ^7^F_1_) and 616.0 nm (^5^D_0_ → ^7^F_2_) (ESI, Fig. S5†) and an Intensity ratio (intensity(5D_0_ → ^7^F_2_)/intensity(^5^D_0_ → ^7^F_1_)), herein after referred to as ^7^F_2_/^7^F_1_ ratio, of 0.5, which is in agreement with the [Eu(H_2_O)_9_]^3+^ complex.^36^ However, since the lifetime of this species is reported with 108 μs, the lifetime of 128 ± 6 μs (ESI, Fig. S6†) indicates the formation of a certain amount of [Eu(NO_3_)(H_2_O)_8_]^2+^ (159 μs).^36^

### Speciation studies of Eu(iii) with C4-BPP in the presence of 2-bromohexanoic acid

**Fig. 8:**
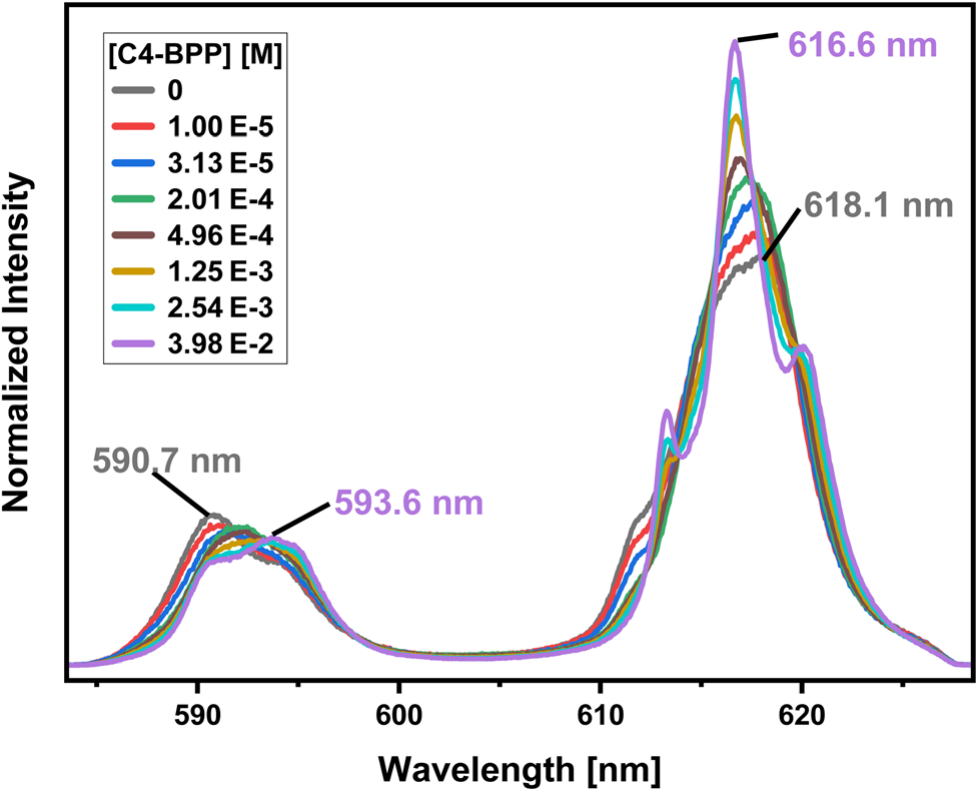
Normalized fluorescence spectra of the ^7^F_1_ and ^7^F_2_ emission bands of Eu(iii) in methanol containing 1.5 vol% water and 5.0 × 10^−1^ mol L^−1^ 2-bromohexanoic acid as a function of the C4-BPP concentration. [Eu(iii)]_ini_ = 1.0 × 10^−5^ mol L^−1^, [C4-BPP] = (0–3.98) × 10^−2^ mol L^−1^. *T* = 293 K.

To investigate the composition of the extracted Eu(iii) species, a titration experiment with C4-BPP in the presence of 2-bromohexanoic acid is performed. Eu(iii) is dissolved in methanol containing 1.5 vol% water and 5.0 × 10^−1^ mol L^−1^ 2-bromohexanoic acid. The concentration of C4-BPP is gradually increased. The normalized emission spectra resulting from the ^5^D_0_ → ^7^F_1_ and ^5^D_0_ → ^7^F_2_ transitions are shown in Fig. 8.

**Fig. 9:**
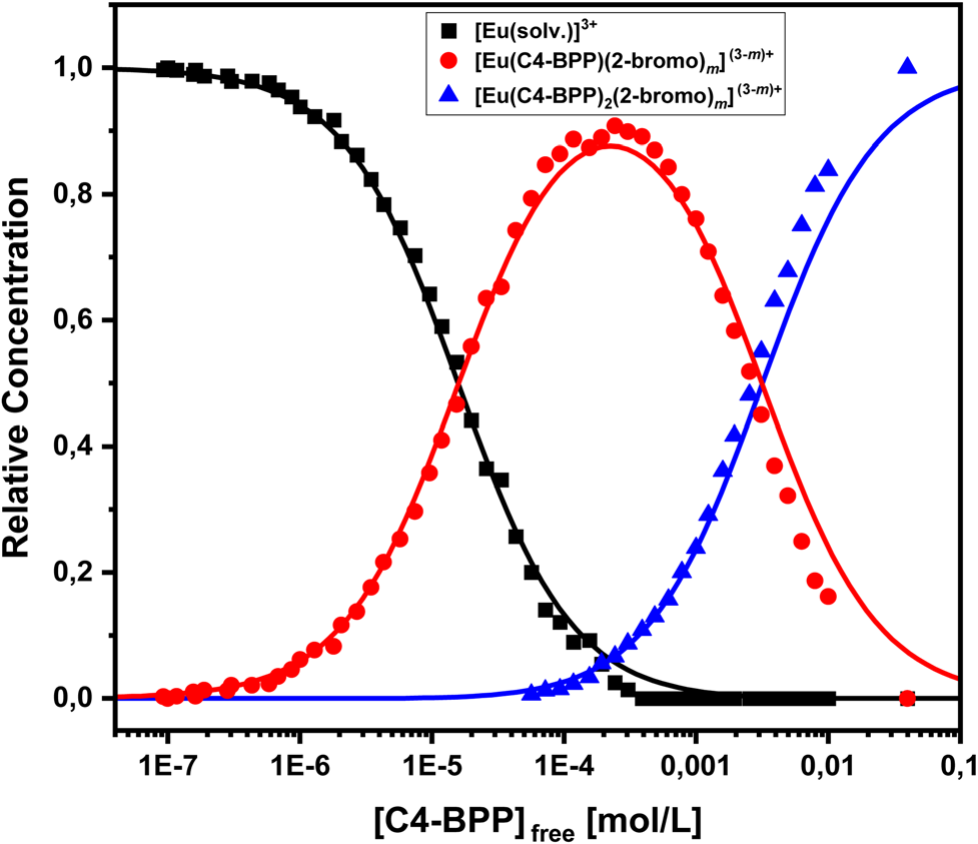
Relative Eu(iii) species concentrations in methanol containing 1.5 vol% water and 5.0 × 10^−1^ mol L^−1^ 2-bromohexanoic acid as a function of the free C4-BPP concentration. Symbols denote experimental data, while lines are calculated using 
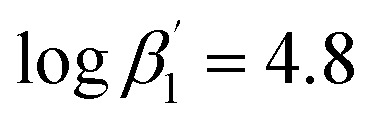
 and 
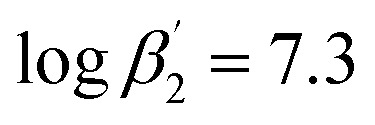
. *T* = 293 K (2-bromohexanoate is abbreviated as 2-bromo).

In absence of C4-BPP the spectrum of Eu(iii) shows two emission bands located at 590.7 nm (^5^D_0_ → ^7^F_1_) and 618.1 nm (^5^D_0_ → ^7^F_2_) (Fig. 8). In contrast to the titration experiment with methanol containing only 1.5 vol% water the ^7^F_2_/^7^F_1_ ratio is significantly higher, around 2.6.^27^ Since the ^5^D_0_ → ^7^F_2_ transition is hypersensitive, this indicates the formation of an asymmetric complex.^37,38^ Thus the formation of [Eu(2-bromohexanoate)_*m*_]^(3−*m*)+^ complexes (*m* = 1–3) are assumed. This is supported by the fluorescence lifetime of 337 ± 17 μs (ESI, Fig. S7†), which is substantially longer than without 2-bromohexanoic acid (212 ± 11 μs).^27^ With increasing C4-BPP concentration the ^5^D_0_ → ^7^F_1_ and the ^5^D_0_ → ^7^F_2_ emission bands change their shape twice. The maxima of the first species are located at 592.1 nm (^5^D_0_ → ^7^F_1_) and 617.6 nm (^5^D_0_ → ^7^F_2_) and of the second species at 593.7 nm (^5^D_0_ → ^7^F_1_) and 616.6 nm (^5^D_0_ → ^7^F_2_). Since Eu(iii) has normally nine coordination sites in total in solution and six of them are occupied with C4-BPP (*n* = 2) the maximum number of 2-bromohexanoates in the inner coordination sphere is three. The variation of the emission spectra in comparison with the ones without 2-bromohexanoic acid shows at least the coordination of one molecule of 2-bromohexanoic acid. Due to the aggregation^23^ of 2-bromohexanoic acid it is only possible to give a range between one and three coordinated molecules. Therefore, the emission spectra are assigned to the [Eu(C4-BPP)_*n*_(2-bromohexanoate)_*m*_]^(3−*m*)+^-complexes (*n* = 1–2, *m* = 1–3), respectively. The ^7^F_2_/^7^F_1_ ratio of the [Eu(C4-BPP)_2_(2-bromohexanoate)_*m*_]^(3−*m*)+^ complex (*m* = 1–3) is 4.8, which indicates a further reduction in symmetry. The Eu(iii) species distribution (Fig. 9) was determined by peak deconvolution of the fluorescence spectra, using the ^5^D_0_ → ^7^F_1_ and ^5^D_0_ → ^7^F_2_ transitions (Fig. 8). The single component spectra (ESI, Fig. S8†) are used to perform the peak deconvolution (for further details about peak deconvolution, see references).^39,40^

The formation of [Eu(C4-BPP)(2-bromohexanoate)_*m*_]^(3−*m*)+^ (*m* = 1–3) starts at a C4-BPP concentration of 5.0 × 10^−7^ mol L^−1^ and reaches a maximum share of 88% at 2.5 × 10^−4^ mol L^−1^. Around this point [Eu(C4-BPP)_2_(2-bromohexanoate)_*m*_]^(3−*m*)+^ (*m* = 1–3) is observable and becomes the dominant species above 3.0 × 10^−3^ mol L^−1^. This complex species shows a fluorescence lifetime of 1473 ± 74 μs (ESI, Fig. S7†) indicating the absence of solvent molecules in the inner coordination sphere. Based on the evolution of the Eu(iii) spectra a stepwise complexation model according to eqn (2) is assumed. To confirm this slope analyses are performed.

2 [M(L)_*n*−1_]^3+^ + L ⇌ [M(L)_*n*_]^3+^ (*n* = 1–3)

3
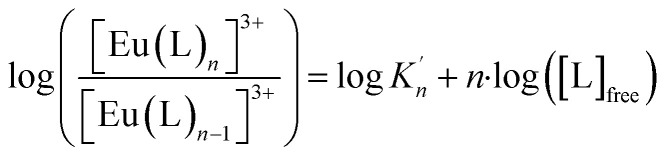


The correlation between the logarithm of [Eu(C4-BPP)_*n*_]^3+^/[Eu(C4-BPP)_*n*−1_]^3+^ (*n* = 1–2) and the logarithm of the free C4-BPP concentration is displayed in the ESI (Fig. S9).†

According to eqn (3) the linear regression yields slopes of 1.03 ± 0.02, and 1.11 ± 0.02 for the formation of the [Eu(C4-BPP)_*n*_(2-bromohexanoate)_*m*_]^(3−*m*)+^ complexes (*n* = 1–2) (*m* = 1–3), respectively (ESI, Fig. S9†). This verifies the assumed complexation model and the correct assignment of the [Eu(C4-BPP)_*n*_(2-bromohexanoate)_*m*_]^(3−*m*)+^ (*n* = 1–2) (*m* = 1–3) complexes. The conditional stability constants are calculated using eqn (4). The obtained values (
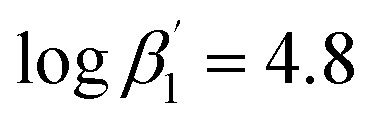
 and 
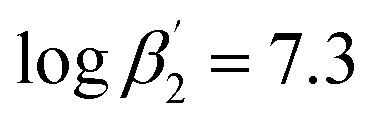
) are smaller than those in the absence of 2-bromohexanoic acid (
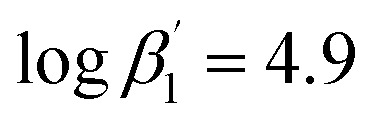
 and 
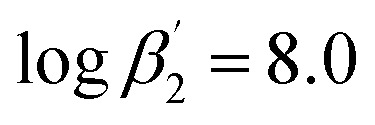
).^27^ This meets the expectation that 2-bromohexanoic acid is a competitive ligand.4
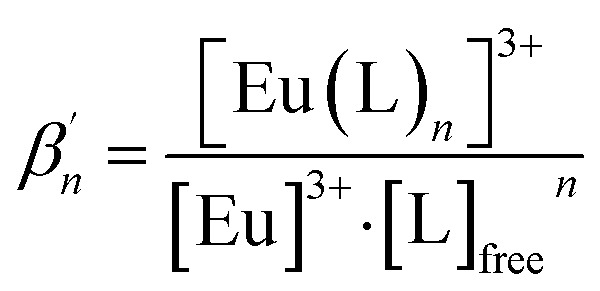


**Fig. 10:**
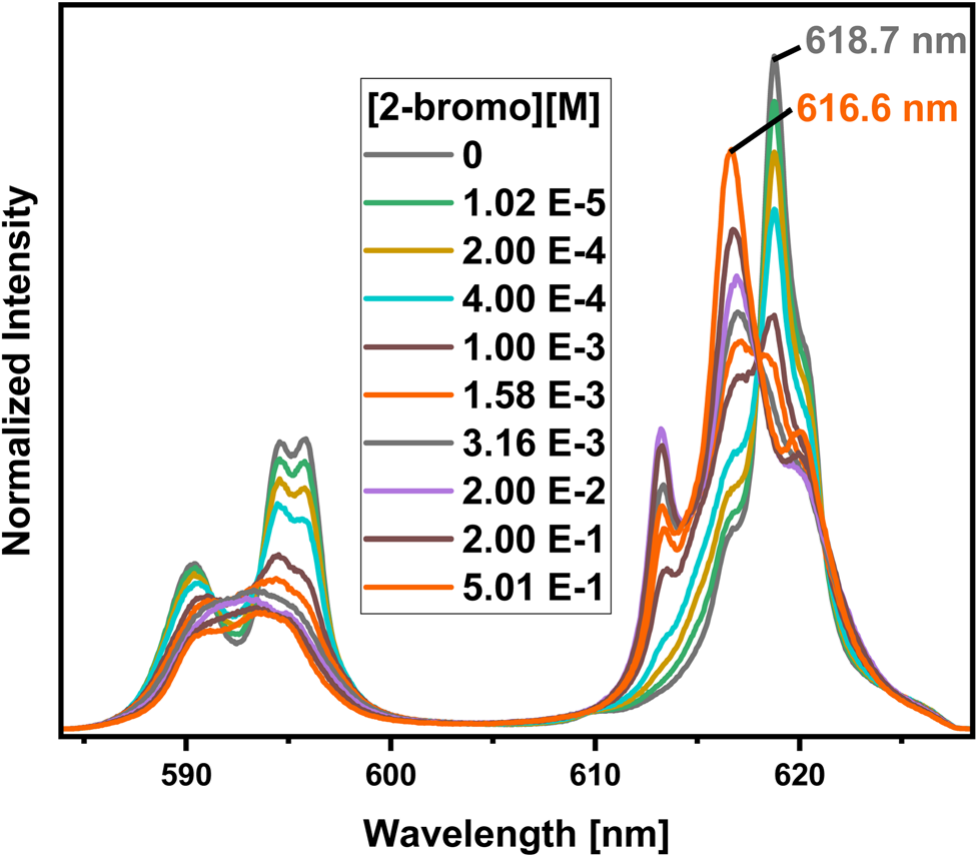
Normalized fluorescence spectra of the ^7^F_1_ and ^7^F_2_ emission bands of Eu(iii) in methanol containing 1.5 vol% water and 3.98 × 10^−2^ mol L^−1^ C4-BPP as a function of the 2-bromohexanoic acid concentration. [Eu(iii)]_ini_ = 1.0 × 10^−5^ mol L^−1^, [2-bromo] = (0–5.01) × 10^−1^ mol L^−1^. *T* = 293 K (2-bromohexanoic acid is abbreviated as 2-bromo).

To further confirm the formation of ternary Eu-C4-BPP-2-bromohexanoate species, a titration series is performed starting with a high concentration of C4-BPP (3.98 × 10^−2^ mol L^−1^) and gradually increasing the concentration of 2-bromohexanoic acid (Fig. 10). In absence of 2-bromohexanoic acid only the complexation of Eu(iii) with C4-BPP is observed with emission bands located at 590.3 nm, 594.6 nm and 595.8 nm (^5^D_0_ → ^7^F_1_) and 618.7 nm (^5^D_0_ → ^7^F_2_). This emission spectrum is assigned to the [Eu(C4-BPP)_3_]^3+^ complex, which is confirmed by the fluorescence lifetime of 2043 ± 103 μs (ESI, Fig. S10†).

With increasing 2-bromohexanoic acid concentration the shape of the emission bands change, indicating the formation of the already known [Eu(C4-BPP)_2_(2-bromohexanoate)_*m*_]^(3−*m*)+^ (*m* = 1–3) complex (Fig. 8 and ESI, Fig. S8†). This species shows a fluorescence lifetime of 1489 ± 75 μs (ESI, Fig. S10†) and a ^7^F_2_/^7^F_1_ ratio of 4.8, which is exactly the same value as for the [Eu(C4-BPP)_2_(2-bromohexanoate)_*m*_]^(3−*m*)+^ (*m* = 1–3) complex (Fig. 8 and ESI, Fig. S8†). Unfortunately, a quantitative analysis of the number of bound 2-bromohexanoic acid ligands is not possible since 2-bromohexanoic acid undergoes associative interaction and thus reducing the free concentration of the lipophilic anion.^23^ Nevertheless, these results show that C4-BPP is unable to form a 1 : 3 complex with Eu(iii) in the presence of 2-bromohexanoic acid, rather forming a ternary complex. This results from a greater steric straining within the complex in comparison to C5-BPP due to the lower flexibility of the ^*t*^Bu moieties (C4-BPP) compared to the neopentyl groups (C5-BPP). This results in lower stability constants of the monoleptic complex in comparison with the ternary complex. Such ternary complexes have already been reported for Cm(iii) with a similar N-donor ligand and 2-bromohexanoic acid.^15^

**Fig. 11:**
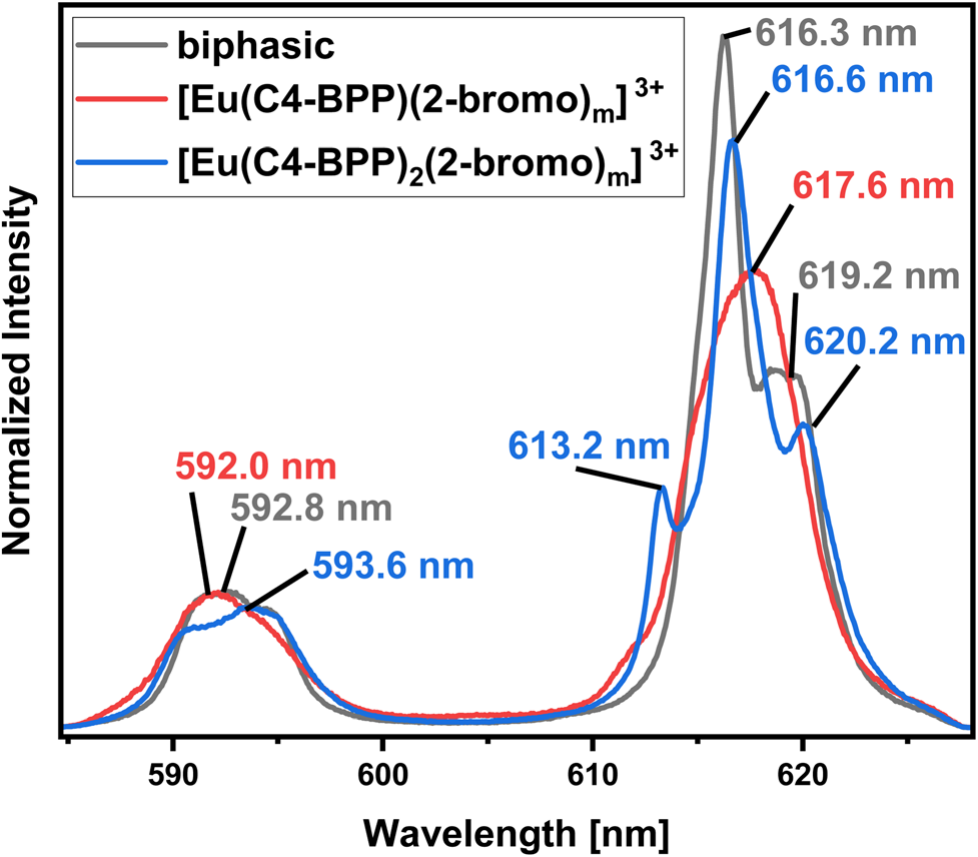
Normalized fluorescence spectra of the ^7^F_1_ and ^7^F_2_ emission bands of Eu(iii) in the organic phase after extraction (black), [Eu(C4-BPP)(2-bromohexanoate)_*m*_]^(3−*m*)+^ (*m* = 1–3) complex (red) and [Eu(C4-BPP)_2_(2-bromohexanoate)_*m*_]^(3−*m*)+^ (*m* = 1–3) complex (blue) in methanol containing 1.5 vol% water and 5.0 × 10^−1^ mol L^−1^ 2-bromohexanoic acid. Organic phase 1.0 × 10^−2^ mol L^−1^ C4-BPP and 5.0 × 10^−1^ mol per L 2-bromohexanoic acid in TPH/1-octanol (10 vol%). Aqueous phase 3.0 × 10^−1^ mol L^−1^ nitric acid. A/O = 1. *T* = 293 K.

Identification of Species in the Solvent Extraction Sample of Eu(iii) in the presence of 2-bromohexanoic acid. Fig. 11 compares the emission spectrum of Eu(iii) extracted into the organic phase (biphasic) to the spectra of the [Eu(C4-BPP)_*n*_(2-bromohexanoate)_*m*_]^(3−*m*)+^ complexes (*n* = 1–2, *m* = 1–3). This shows good agreement of the emission spectrum of the extracted species with that of the [Eu(C4-BPP)_2_(2-bromohexanoate)]^(3−*m*)+^ complex (*m* = 1–3). Furthermore, the fluorescence lifetime of the extracted complex (1314 ± 66 μs) fits with the lifetime of the [Eu(C4 BPP)_2_(2-bromohexanoate)_*m*_]^(3−*m*)+^ (*m* = 1–3) complex (1473 ± 74 μs). Thus the extracted species is identified as the ternary [Eu(C4-BPP)_2_(2-bromohexanoate)_*m*_]^(3−*m*)+^ (*m* = 1–3) complex. The small deviation in shape may result from the absence of nitrate in the titration series and TPH instead of methanol as a solvent.

## Conclusion

In continuation of previous investigations of the complexation behavior of C4-BPP,^27^ the focus of this study was primarily to understand its behavior under extraction conditions.

C4-BPP (in presence of 2-bromohexanoic acid acting as a lipophilic anion source) extracts Am(iii) with excellent selectivity over Ln(iii) from nitric acid concentrations <0.3 mol L^−1^. Even though Nd(iii) possesses the highest distribution ratio among the Ln(iii) series a separation factor of SF_Am(III)/Nd(III)_ ≈ 60 allows a sufficient separation of Am over Nd and thus all Ln(iii). A separation factor up to SF_Am(III)/Eu(III)_ ≈ 200 is achieved, which is considerably higher than that obtained for the structurally related C5-BPP (SF_Am(III)/Eu(III)_ ≈ 100).

To determine the origin of these excellent properties in solvent extraction fundamental speciation studies using TRLFS were performed. The results confirm that Cm(iii) forms 1 : 3 complexes with C4-BPP during extraction, which is in agreement with the results obtained for C5-BPP.^21^ In contrast, Eu(iii) is extracted in the form of a ternary complex, [Eu(C4-BPP)_2_(2-bromohexanoate)_*m*_]^(3−*m*)+^ (*m* = 1–3) due to the greater steric straining within the monoleptic 1 : 3 complex. The absence of [Eu(C4-BPP)_3_]^3+^results in larger differences of the stability constants of the Cm(iii) and Eu(iii) complexes of C4-BPP in comparison with the corresponding C5-BPP-complexes. Thus a significantly higher separation factor is observable for C4-BPP.

The selectivity for An(iii) over Ln(iii) of heterocyclic N-donor extracting agents is generally driven by differences in the stability constants of the respective complexes. The few studies comparing the structures of these complexes formed upon extraction clearly show isostructural An(iii) and Ln(iii) complexes. To our knowledge, the present study is the first one reporting distinct structural differences to explain selectivity, with Cm(iii) being extracted in the form of a homoleptic 1 : 3 complex and Eu(iii) in the form of a heteroleptic complex containing both C4-BPP and bromohexanoate anion(s) in the inner coordination sphere.

## Data availability

Data for this article, including [TRLFS raw data] are available at [Open Science Framework] at [https://doi.org/10.17605/OSF.IO/7GJHY].

## Conflicts of interest

No potential conflict of interest was reported by the author(s).

## Supplementary Material

RA-014-D4RA05630B-s001
