# Supplementary material for: Extraction and complexation studies with 2,6-bis(5-(tert-butyl)-1H-pyrazol-3-yl)pyridine in the presence of 2-bromohexanoic acid
Source: RSC Adv. 2024 Sep 5;14(39):28415–22. doi: 10.1039/d4ra05630b (PMC11376233; doi:10.1039/d4ra05630b)
Supplement: RA-014-D4RA05630B-s001 [file RA-014-D4RA05630B-s001.pdf]

Supplementary Information (SI) for RSC Advances.
This journal is © The Royal Society of Chemistry 2024

**Supplementary Information for**

# Extraction and Complexation studies with 2,6-bis(5-(tert-butyl)-1*H*-pyrazol-3-yl)pyridine in the presence of 2-bromohexanoic acid.

Jonas Stracke,[a,b] Patrik Weßling,[b] Thomas Sittel,[a] Paul Meiners,[b] Andreas Geist,[a] and Petra J. Panak.[a,b]

[a]Karlsruhe Institute of Technology (KIT), Institute for Nuclear Waste Disposal (INE), P.O. Box 3640, 76021 Karlsruhe, Germany

[b]Heidelberg University, Institut für Physikalische Chemie, Im Neuenheimer Feld 253, 69120 Heidelberg, Germany

KEYWORDS Actinides, Lanthanides, Lipophilic ligands, Complexation, Selectivity, N-Donor ligand.

Corresponding Authors:

Jonas Stracke: Jonas.stracke@kit.edu

# Table of contents

TRLFS Cm(III)........................................................................................................................ 3
TRLFS Eu(III)........................................................................................................................ 5

# TRLFS Cm(III)

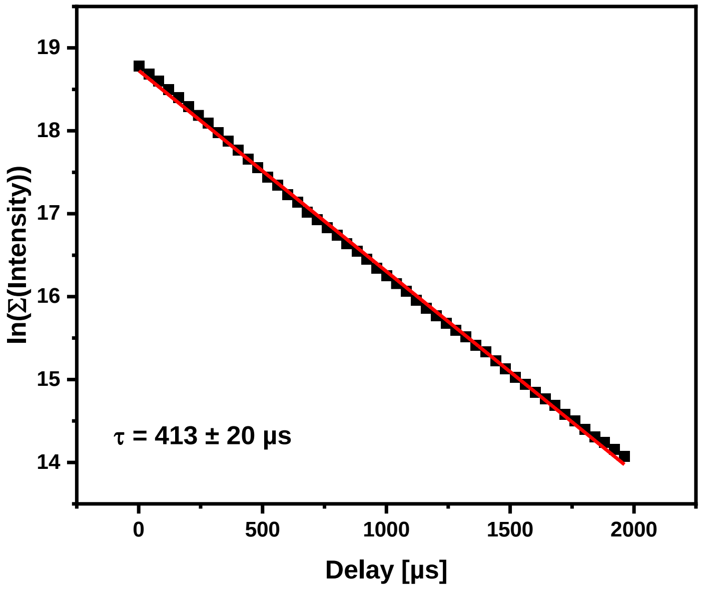


**Figure S1:** Fluorescence lifetime of $[Cm(C4\text{-}BPP)_3]^{3+}$ in the organic phase after extraction. Organic phase $1.0 \times 10^{-2}$ mol/L C4-BPP and $5.0 \times 10^{-1}$ mol/L 2-bromohexanoic acid in TPH/1-octanol (10 vol.%). Aqueous phase $3.0 \times 10^{-1}$ mol/L nitric acid. A/O = 1. T = 293 K.

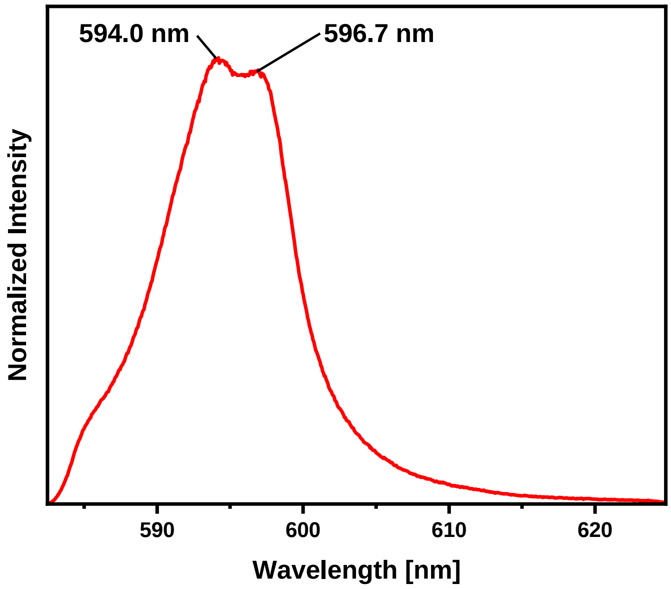


**Figure S2:** Normalized fluorescence spectra of Cm(III) in the aqueous phase after extraction. Organic phase $1.0 \times 10^{-2}$ mol/L C4-BPP and $5.0 \times 10^{-1}$ mol/L 2-bromohexanoic acid in TPH/1-octanol (10 vol.%). Aqueous phase $3.0 \times 10^{-1}$ mol/L nitric acid. A/O = 1. T = 293 K.

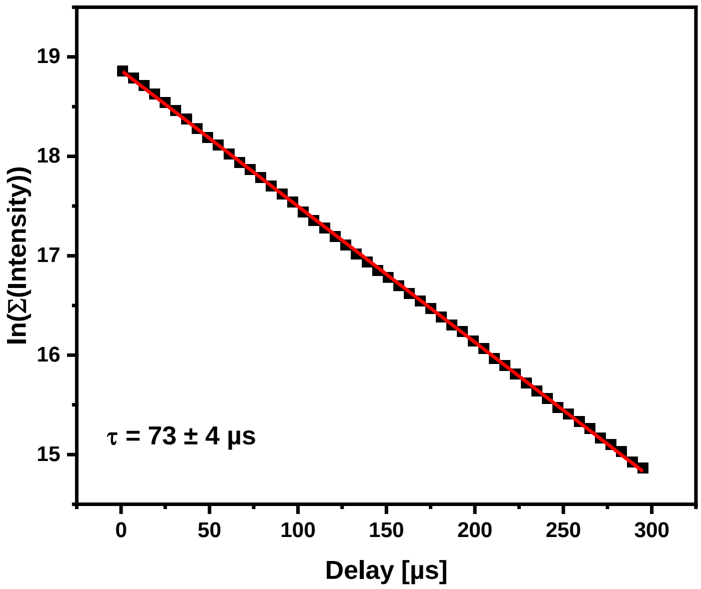


**Figure S3:** Fluorescence lifetime of $[Cm(solv.)]^{3+}$ in the aqueous phase after extraction. Organic phase $1.0 \times 10^{-2}$ mol/L C4-BPP and $5.0 \times 10^{-1}$ mol/L 2-bromohexanoic acid in TPH/1-octanol (10 vol.%). Aqueous phase $3.0 \times 10^{-1}$ mol/L nitric acid. A/O = 1. T = 293 K.

# TRLFS Eu(III)

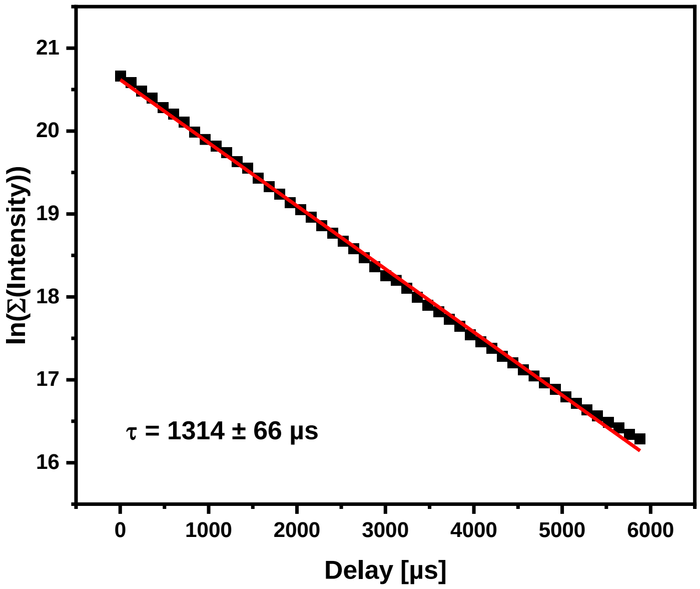


**Figure S4:** Fluorescence lifetime of [Eu(C4-BPP)$_2$(2-bromohexanoate)$_m$]$^{(3-m)+}$ ($m$ =1-3) in the organic phase after extraction. Organic phase 1.0 × 10$^{-2}$ mol/L C4-BPP and 5.0 × 10$^{-1}$ mol/L 2-bromohexanoic acid in TPH/1-octanol (10 vol.%). Aqueous phase 3.0 × 10$^{-1}$ mol/L nitric acid. A/O = 1. T = 293 K.

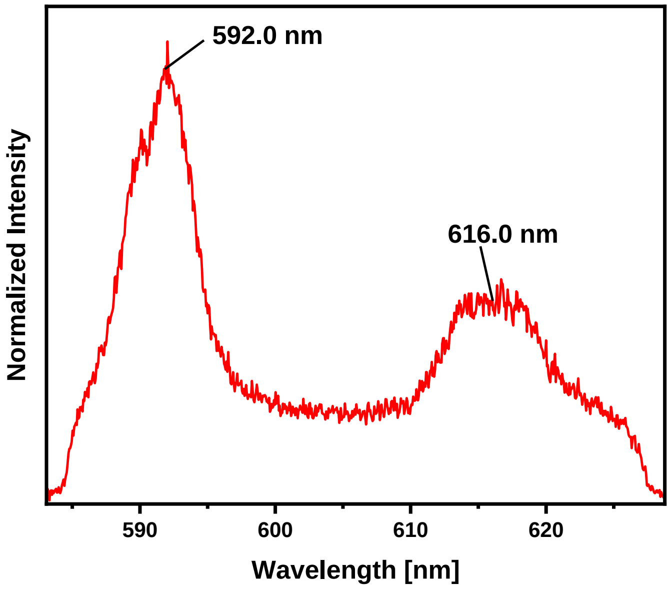


**Figure S5:** Normalized fluorescence spectra of the $^7F_1$ and $^7F_2$ emission bands of Eu(III) in the aqueous phase after extraction. Organic phase 1.0 × 10$^{-2}$ mol/L C4-BPP and 5.0 × 10$^{-1}$ mol/L 2-bromohexanoic acid in TPH/1-octanol (10 vol.%). Aqueous phase 3.0 × 10$^{-1}$ mol/L nitric acid. A/O = 1. T = 293 K.

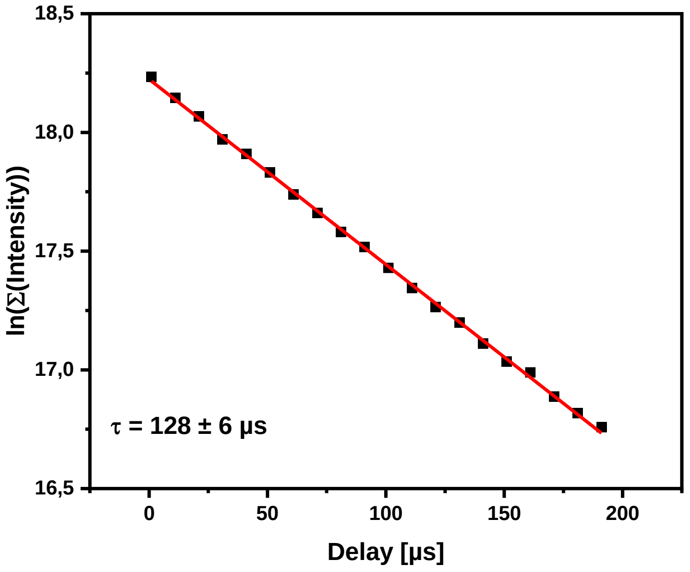


**Figure S6:** Fluorescence lifetime of $[Eu(solv.)]^{3+}$ in the aqueous phase after extraction. Organic phase $1.0 \times 10^{-2}$ mol/L C4-BPP and $5.0 \times 10^{-1}$ mol/L 2-bromohexanoic acid in TPH/1-octanol (10 vol.%). Aqueous phase $3.0 \times 10^{-1}$ mol/L nitric acid. A/O = 1. T = 293 K.

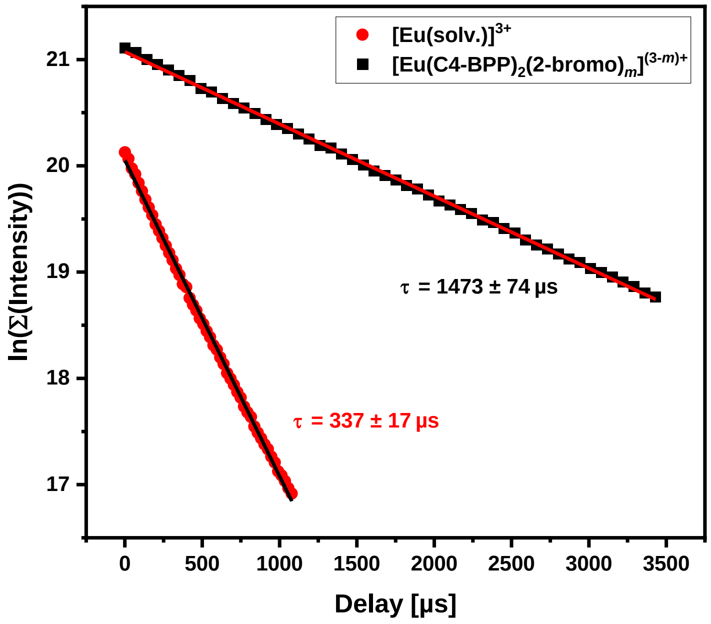


**Figure S7:** Fluorescence lifetime of $[Eu(C4\text{-}BPP)_n(2\text{-bromohexanoate})_m]^{(3-m)+}$ ($n$ = 0, 2; $m$ = 1-3) in methanol containing 1.5 vol.% water and 0.5 mol/L 2-bromohexanoic acid. [C4-BPP] = 0 mol/L ($n$ = 0), $1.00 \times 10^{-2}$ mol/L ($n$ = 2), $[Eu]_{ini} = 1.0 \times 10^{-5}$ mol/L. (2-bromohexanoate is abbreviated as 2-bromo).

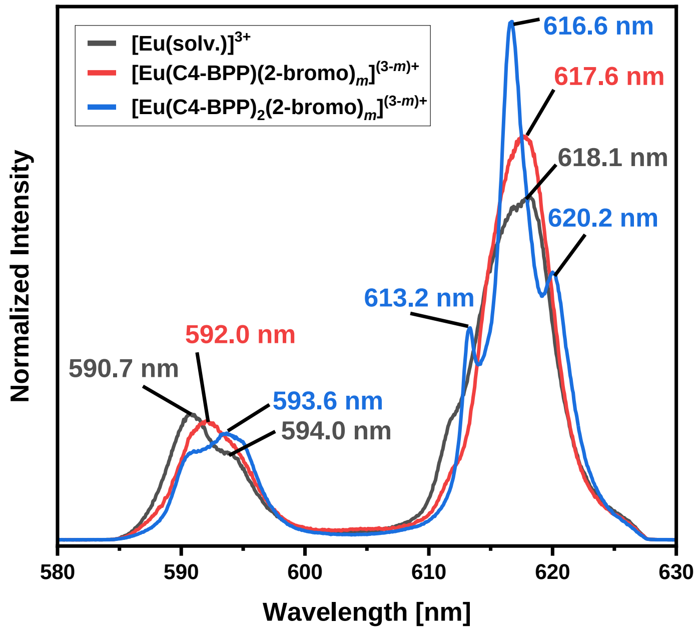


**Figure S8:** Normalized single component spectra of the $[Eu(C4\text{-}BPP)_n(2\text{-bromohexanoate})_m]^{(3-m)+}$ ($n$ = 0-2, $m$ = 1-3) complexes in methanol containing 1.5 vol.% water and $5.0 \times 10^{-1}$ mol/L 2-bromohexanoic acid. $[Eu(III)]_{ini} = 1.0 \times 10^{-5}$ mol/L. (2-bromohexanoate is abbreviated as 2-bromo).

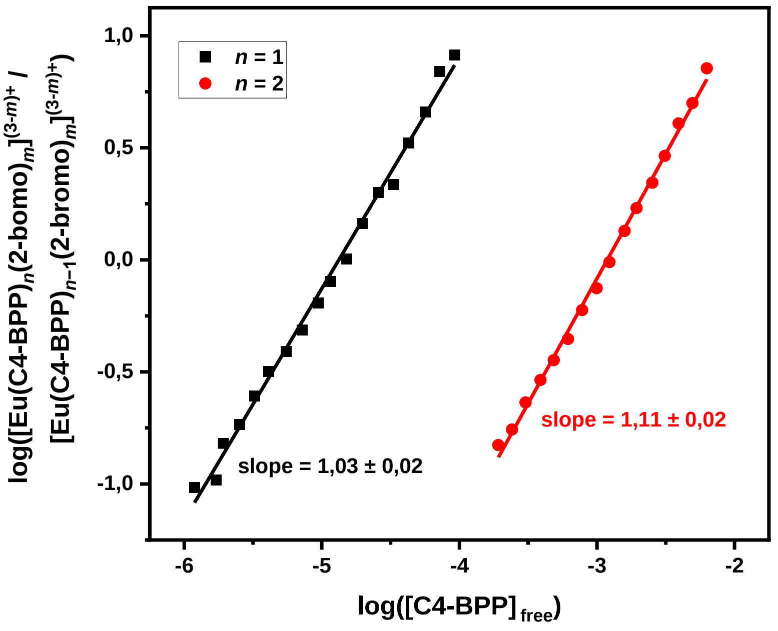


**Figure S9:** Slope analyses for the complexation of Eu(III) with C4-BPP in methanol containing 1.5 vol.% water and $5.0 \times 10^{-1}$ mol/L 2-bromohexanoic acid. Plot of log([Eu(C4-BPP)$_n$(2-bromohexanoate)$_m$]$^{(3-m)+}$/[Eu(C4-BPP)$_{n-1}$(2-bromohexanoate)$_m$]$^{(3-m)+}$) ($m$ =1-3) vs. the logarithm of the free C4-BPP concentration (2-bromohexanoate is abbreviated as 2-bromo).

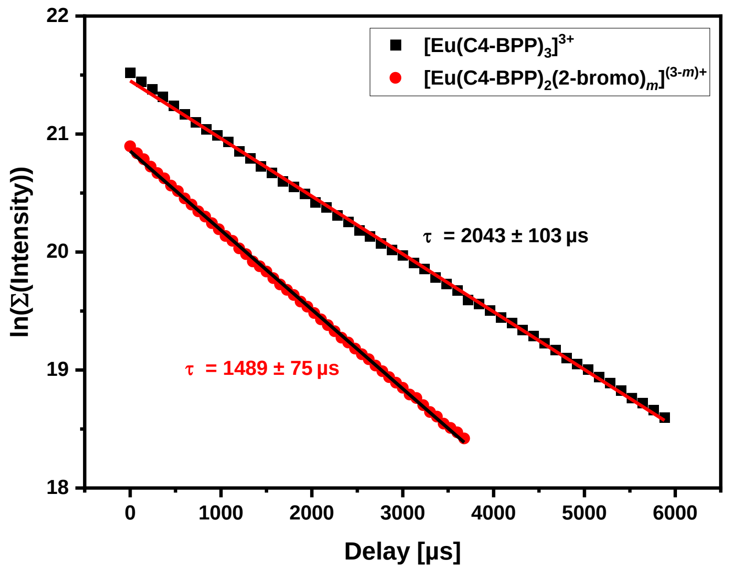


**Figure S10:** Fluorescence lifetime of $[Eu(C4\text{-}BPP)_n(2\text{-bromohexanoate})_m]^{(3-m)+}$ ($n$ = 2, 3; $m$ = 0-3) in methanol containing 1.5 vol.% water and $3.98 \times 10^{-2}$ mol/L C4-BPP. [2-bromohexanoic acid] = 0 mol/L ($n$ = 0), $5.01 \times 10^{-1}$ mol/L ($n$ = 2), $[Eu]_{ini} = 1.0 \times 10^{-5}$ mol/L. (2-bromohexanoate is abbreviated as 2-bromo).
